# Single Commercially Available IC-Based Electronically Controllable Voltage-Mode First-Order Multifunction Filter with Complete Standard Functions and Low Output Impedance

**DOI:** 10.3390/s21217376

**Published:** 2021-11-06

**Authors:** Winai Jaikla, Unchittha Buakhong, Surapong Siripongdee, Fabian Khateb, Roman Sotner, Phamorn Silapan, Peerawut Suwanjan, Amornchai Chaichana

**Affiliations:** 1 Department of Engineering Education, School of Industrial Education and Technology, King Mongkut’s Institute of Technology Ladkrabang, Bangkok 10520, Thailand; 64603063@kmitl.ac.th (U.B.); surapong.si@kmitl.ac.th (S.S.); peerawut.su@kmitl.ac.th (P.S.); amornchai.ch@kmitl.ac.th (A.C.); 2 Department of Microelectronics, Brno University of Technology, Technická 10, 601 90 Brno, Czech Republic; khateb@vutbr.cz; 3 Department of Information and Communication Technology in Medicine, Czech Technical University in Prague, Nám. Sítná 3105, 272 01 Kladno, Czech Republic; 4 Department of Radio Electronics, Brno University of Technology, 12, 616 00 Brno, Czech Republic; sotner@feec.vutbr.cz; 5 Department of Electrical Engineering, Faculty of Engineering and Industrial Technology, Silpakorn University, Nakornpathom 73000, Thailand; silapan_p@su.ac.th

**Keywords:** multifunction filter, LT1228, electronic control, voltage-mode, first-order circuit, phase shifted circuit, active building block

## Abstract

This paper presents the design of a voltage-mode three-input single-output multifunction first-order filter employing commercially available LT1228 IC for easy verification of the proposed circuit by laboratory measurements. The proposed filter is very simple, consisting of a single LT1228 as an active device with two resistors and one capacitor. The output voltage node is low impedance, resulting in an easy cascade-ability with other voltage-mode configurations. The proposed filter provides four filter responses: low-pass filter (LP), high-pass filter (HP), inverting all-pass filter (AP−), and non-inverting all-pass filter (AP+) in the same circuit configuration. The selection of output filter responses can be conducted without additional inverting or double gains, which is easy to be controlled by the digital method. The control of pole frequency and phase response can be conducted electronically through the bias current (*I_B_*). The matching condition during tuning the phase response with constant voltage gain is not required. Moreover, the pass-band voltage gain of the LP and HP functions can be controlled by adjusting the value of resistors without affecting the pole frequency and phase response. Additionally, the phase responses of the AP filters can be selected as both lagging or leading phase responses. The parasitic effects on the filtering performances were also analyzed and studied. The performances of the proposed filter were simulated and experimented with a ±5 V voltage supply. For the AP+ experimental result, the leading phase response for 1 kHz to 1 MHz frequency changed from 180 to 0 degrees. For the AP− experimental result, the lagging phase response for 1 kHz to 1 MHz frequency changed from 0 to −180 degrees. The design of the quadrature oscillator based on the proposed first-order filter is also included as an application example.

## 1. Introduction

In sensor applications, an active filter plays a very importance role, for example, in the electrocardiographic (ECG) system [1], phase sensitive detection [2], biosensors [3], etc. It is frequency employed to detect the wanted signal in these applications. The synthesis and design of the filter using an active building block (ABB) to obtain new active circuits has received prominent attention [4,5,6,7]. The use of active building blocks in the circuit design leads to a compact structure with less passive elements. Some active circuits based on the active building block are cascade-able without requiring additional buffer devices. In addition, the circuit parameters of the active circuit realized from the electronically controllable active building block are easily controlled by the microcontroller, which is essential for modern analog signal processing circuits [8,9,10].

Although the design of the circuits to be implemented into an integrated circuit provides many advantages, for example, high circuit efficiency, small size, low voltage, low power, etc. However, the implementation of an integrated circuit is quite costly. This will be cost-effective for mass production. Therefore, the use of a commercially available active building block in circuit design for use in specific applications is an attractive and cost-effective alternative [11,12]. The analog circuits realized from the commercially available active building block have been continuously introduced in the open literature [13,14,15].

**Figure 1:**
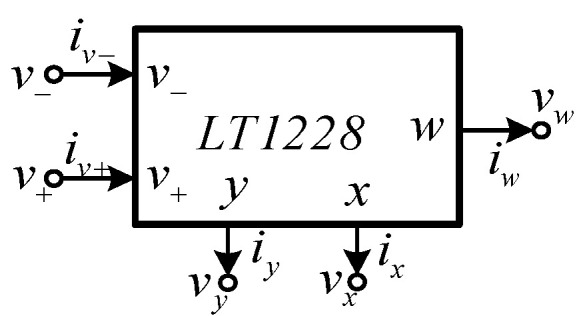
Symbol notation of LT1228.

**Figure 2:**
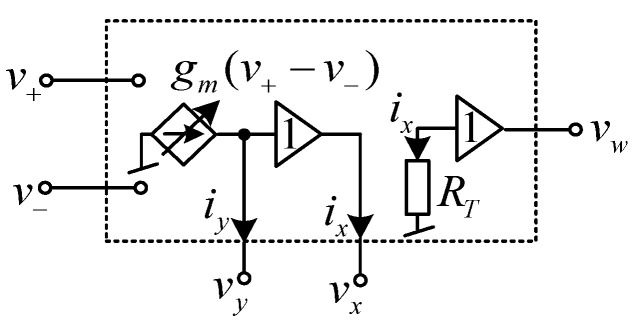
Ideal equivalent circuit of LT1228.

The realization of first-order filters using active building blocks has drawn great attention [15,16,17,18,19,20,21,22,23,24,25,26,27,28,29,30,31,32,33,34,35,36,37,38,39,40,41,42,43,44,45]. Generally, the first-order configuration can provide three standard filtering responses: low-pass (LP), high-pass (HP), and all-pass (AP) functions. The all-pass filter is used to shift the phase of an output signal with a constant amplitude over the entire frequency band. If the phase response of the all-pass filter is considered, there are two kinds of all-pass filter: the lagging and leading phase shifters. These phase shifters are also called the inverting and non-inverting all-pass filters, respectively. The first-order filters that function only with the phase shifter are proposed in [15,16,17,18,19,20]. The universal or multifunction first-order filters that perform multiple filtering functions in the same structure have been proposed in [21,22,23,24,25,26,27,28,29,30,31,32,33,34,35,36,37,38,39,40,41,42,43,44,45]. Most universal first-order filters [21,23,24,26,29,30,31] (Figure 1), (Figure 2) [34,35,36,37,39,41,45] are realized in current-mode (CM) configuration, which can avoid the use of additional summing or subtracting circuits. With this feature, the current-mode circuit enjoys a compact structure. Transresistance-mode (RM) and transconductance-mode (TM) universal first order filters are reported in [22,28,31] (Figure 2), respectively. The universal first-order filters in voltage-mode (VM) configuration are proposed in [23,25,27,32,33,38,40,42,43,44]. The comparison between the proposed first-order universal filter and the previous ones presented in [21,22,23,24,25,26,27,28,29,30,31,32,33,34,35,36,37,38,39,40,41,42,43,44,45] is summarized in Table 1. From the literature survey in Table 1, the following conclusions were established:

- Most of the proposed universal first-order filters are emphasized for the on-chip realization of both CMOS [21,22,23,24,25,26,27,28,29,30,31,32,34,35,36,40,41,45] or BJT [33,37,39] technology. As stated above, the implementation of an on-chip circuit is quite costly. Although the CMOS-based filters in [21,30,31,32,45] can be realized using the commercially available ICs, they require a lot of ICs. The commercial IC based first-order filters are reported in [38,42,43,44]. However, the filters in [38,42,44] used five, three, and two commercially available ICs, respectively. Additionally, the filter in [42] requires four passive resistors and that in [43] uses six passive resistors.
- The realization of a current-mode circuit is a compact structure and can avoid the use of additional summing or subtracting circuits at the output node. However, the current-mode universal filters in [21,23] (Figure 2) [24,26,29,30,31,34,35,36,37,38,39,41] use the active building block, which has multiple output current terminals. These filters will provide high performances when they are implemented into an integrated circuit, which is quite costly.
- Most of the universal first-order filters shown in Table 1 can provide three responses: low-pass, high-pass, and all-pass functions (except in [22], which gives only two filtering responses). However, the lagging and leading phase responses of the all-pass filters in [21,22,23,24,25,26,27,28,29,30,31,33,36,38,40,41,42,43] are not given in the same circuit structure.
- In practice, if the input signal magnitude of the filter is low, the pass-band gain of the filters should be tunable. Therefore, the gain controllable active filter is needed to avoid using an additional amplifier. However, the pass-band gain of the filters in [21,23,24,26,29,30,31] (Figure 1) [32,34,35,36,38,39,40,41] are not controllable.
- The pole frequency and phase shift angle of the filters in [23,25,29,32,34,40,41,42] are not electronically controlled. Although the filters in [21,24,35] are electronically controllable, the passive resistor was replaced by the MOS transistor to achieve electronic controllability, which provides a narrow tuning range.
- During the tuning phase response, simultaneously adjusting two or three parameters in zero and pole frequency is required [24,25,29,30,40,41].
- To avoid the use of additional buffer devices at the output node of the filter, the voltage output node should be low impedance and the current output node should be high impedance.

The aim of this paper was to realize the universal filter by employing a single commercially available IC, LT1228 (Linear Technology, Milpitas, CA, US), as an active device. The rest of this paper is as follows: the principle of operation is shown in Section 2, containing an overview of LT1228, the proposed filter, and study of parasitic effects. Section 3 shows the simulation and experimental results. The application example of the quadrature oscillator is described in Section 4. Finally, a brief conclusion is shown in Section 5.

## 2. Principle of Operation

### 2.1. Overview of LT1228

The first order multifunction filter considered in this paper was based on the use of a commercially available IC, LT1228 [43]. Before embarking on the description of the proposed filter, the characteristics of LT1228 will be described. The LT1228 is a monolithic integrated circuit (IC), which is commercially manufactured by Linear Technology Corporation. This IC is an 8-pin DIP package. For easy consideration, the electrical symbolic representation of LT1228 is drawn in Figure 1, where *v_+_* and *v*_−_ are voltage input terminal; *y* is both the current output and voltage input terminal; *x* is also both voltage output and current input terminal; and *w* is voltage output terminal. Output terminal, y and input terminals, *v_+_* and *v*_−_ have infinite internal impedance, while *x* and *w* terminals have low internal impedance. The equivalent representation of LT1228 is depicted in Figure 2. The ideal terminal relations of LT1228, as shown in Figure 1, can be characterized with the following matrix equation

(1) $$\left(\begin{array}{c} i_{v+}\\ i_{v-}\\ i_{y}\\ v_{x}\\ v_{w} \end{array}\right)=\left(\begin{array}{ccccc} 0 & 0 & 0 & 0 & 0\\ 0 & 0 & 0 & 0 & 0\\ g_{m} & -g_{m} & 0 & 0 & 0\\ 0 & 0 & 1 & 0 & 0\\ 0 & 0 & 0 & R_{T} & 0 \end{array}\right)\left(\begin{array}{c} v_{+}\\ v_{-}\\ v_{y}\\ i_{x}\\ i_{w} \end{array}\right),$$

*R_T_* represents the transresistance gain of LT1228. Ideally, *R_T_* is an infinite resistance. Therefore, LT1228 will have infinite open-loop voltage gain. *g_m_* represents the transconductance gain, which is controlled by an external DC bias current (*I_B_*) as follows

(2) $$g_{m}≅\frac{I_{B}}{3.87V_{T}}.$$

Here, *V_T_* is the thermal voltage. As shown in Equation (2), the *g_m_* is electronically controllable, thus the LT1228 based circuits are easily controlled by a microcomputer or microcontroller.

### 2.2. Proposed First Order Multifunction Filter Using Single LT1228

**Figure 3:**
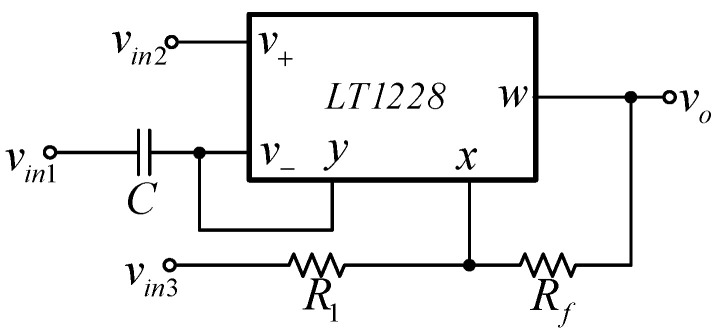
Proposed voltage-mode first-order multifunction filter.

The proposed first order multifunction filter is illustrated in Figure 3. The proposed filter is formed by one LT1228, one capacitor, and two resistors. It was found that the proposed filter using only one commercially available IC, which was easier and cheaper to verify the circuit performances by laboratory measurements than the non-commercially available IC-based circuits. The proposed filter has three voltage input nodes, named *v_in_*_1_, *v_in_*_2_, and *v_in_*_3_ with single voltage output node, *v_o_*. The voltage output node is at the *w* terminal of LT1228, which ideally offers zero output impedance. With this advantage, the proposed filter can be connected to external loads or the input node of other circuits without using additional buffer devices. However, in practice, the output resistance at the *w* terminal (*r_w_*) is not zero, thus the output resistance (*z_o_*) of the proposed filter is around *z_o_* ≅ *r_w_*//*R_f_*. A straightforward analysis of the first-order multifunction circuit in Figure 3 gives the following output voltage, *v_o_*

(3) $$v_{o}=\frac{s\frac{C}{g_{m}}\left(1+\frac{R_{f}}{R_{1}}\right)v_{in1}+\left(1+\frac{R_{f}}{R_{1}}\right)v_{in2}-\frac{R_{f}}{R_{1}}\left(s\frac{C}{g_{m}}+1\right)v_{in3}}{s\frac{C}{g_{m}}+1}.$$

From Equation (3), it can be found that four standard first-order filtering functions—low-pass, high-pass, non-inverting all-pass, and inverting all-pass responses—can be obtained by applying the input signal to the appropriate input voltage nodes, *v_in_*_1_, *v_in_*_2_, and *v_in_*_3_. The selection for each filter response is given in Table 2, where the number 1 represents applying the input signal to that input node and the number 0 represents connecting that input node to ground. The filtering parameters are also given in Table 2.

It was found from Table 2 that the selection of output filter responses can be carried out without additional circuits and is easily controlled by a digital method using a microcontroller or microcomputer. In addition, when a specific switching system or amplifier/multipath gain control is used, a reconfigurable feature (reconnection-less change of transfer response) will be obtained. The proposed filter can provide four filter responses: LP filter, HP filter, inverting AP− filter, and non-inverting AP+ filter in the same circuit configuration. The control of pole frequency and phase response can be conducted electronically. The matching condition during tuning the phase response with constant voltage gain is not required. Moreover, the pass-band voltage gain of the LP and HP functions can be controlled by adjusting the value of resistors without affecting the pole frequency and phase response. Additionally, the phase response of the all-pass filter can be selected as both lagging or leading phase responses. However, the temperature variation affects the natural frequency and phase responses of the proposed filter.

### 2.3. Study of Parasitic Effects

**Figure 4:**
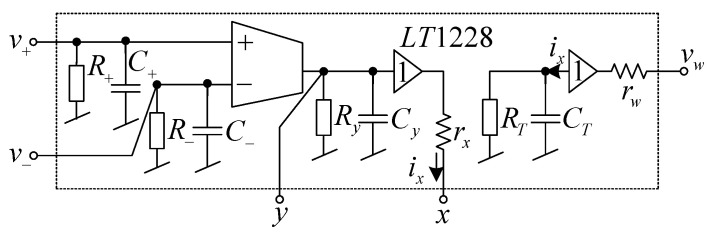
The LT1228 circuit model with parasitic elements.

**Figure 5:**
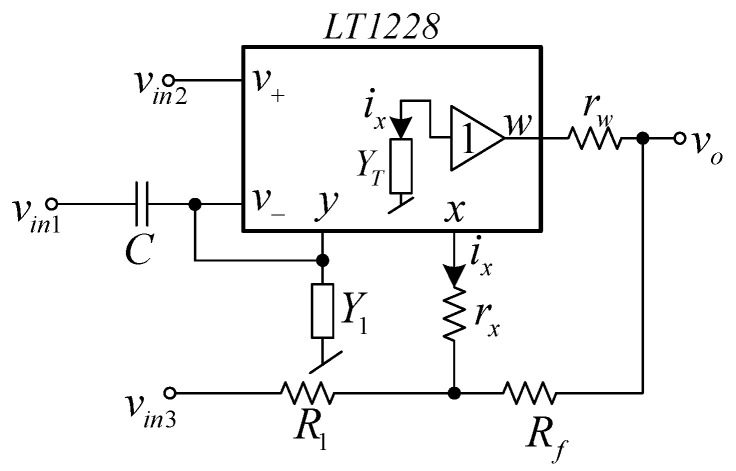
Proposed filter with parasitic elements.

The influence of the parasitic elements on the filtering performances are studied in this section. The LT1228 circuit model with parasitic impedances is drawn in Figure 4. With these parasitic elements, the proposed filter can be modeled as shown in Figure 5. Performing the circuit analysis of the circuit in Figure 5 yields the following output voltage

(4) $$v_{o}=\frac{\left[s\frac{C}{g_{m}}\left(\frac{R_{f}}{R_{1}}+1+Y_{T}r_{w}\right)v_{in1}+\left(\frac{R_{f}}{R_{1}}+1+Y_{T}r_{w}\right)v_{in2}-\frac{R_{f}}{R_{1}}\left(1-\frac{Y_{T}r_{x}r_{w}}{R_{f}}\right)\left(s\frac{C^{*}}{g_{m}}+\frac{G^{*}}{g_{m}}+1\right)v_{in3}\right]}{\left[1+Y_{T}\left(\frac{r_{w}r_{x}}{R_{1}}+r_{w}+\frac{R_{f}r_{x}}{R_{1}}+r_{x}+R_{f}\right)\right]\left(s\frac{C^{*}}{g_{m}}+\frac{G^{*}}{g_{m}}+1\right)}.$$

where $C^{*}=C+C_{-}+C_{y}$ and $G^{*}=G_{-}+G_{y}$. It is found from Equation (4) that the parasitic elements *Y_T_* (*sC_T_* + *G_T_*), *r_x_* and *r_w_* appear on both zero and pole. The parasitic resistances *R_T_*, *r_x_*, and *r_w_* affect the pass-band gain at 0 Hz. The pole that limits the operational frequency (at high frequency) or bandwidth of the proposed circuit is determined from the first term of the denominator of Equation (4). If *r_x_*, *r_w_* ≅ 0, and *R_T_* >> *R*_1_, *R_f_*, the operational frequency of the proposed filter is determined by

(5) $$f_{op}≅\frac{1}{2\pi C_{T}R_{f}}.$$

It also provides interesting information that a small value of resistor *R_f_* can increase the operating frequency of the proposed circuit. If *r_x_*, *r_w_* ≅ 0, and *R_T_* >> *R*_1_, *R_f_* and the operational frequency is less than 1/2π*C_T_R_f_*, the output voltage in Equation (4) is approximated to

(6) $$v_{o}≅\frac{s\frac{C}{g_{m}}\left(\frac{R_{f}}{R_{1}}+1\right)v_{in1}+\left(\frac{R_{f}}{R_{1}}+1\right)v_{in2}-\frac{R_{f}}{R_{1}}\left(s\frac{C^{*}}{g_{m}}+\frac{G^{*}}{g_{m}}+1\right)v_{in3}}{s\frac{C^{*}}{g_{m}}+\frac{G^{*}}{g_{m}}+1}.$$

From Equation (6), the filtering parameters with parasitic effect are given in Table 3.

## 3. Simulation and Experimental Results

**Figure 6:**
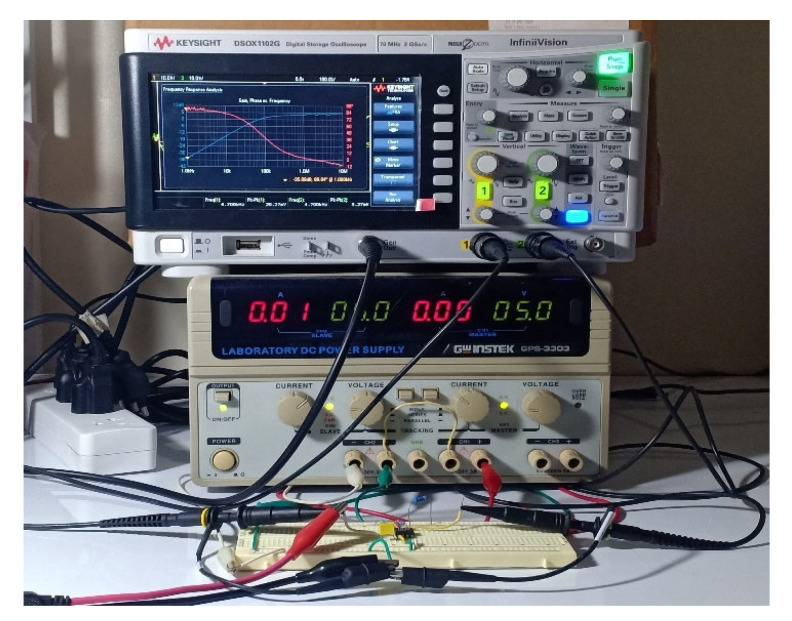
Experimental setup.

**Figure 7:**
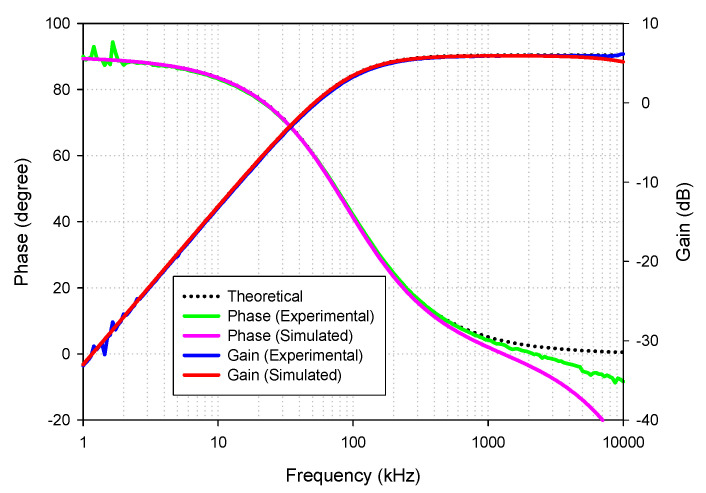
Frequency gain and phase response of HP.

To verify the functionality of the proposed circuit, the Pspice simulation using the LT1228 Pspice macro model (level 3) and experiments using the commercially available LT1228 IC were carried out. The Keysight DSOX-1102G oscilloscope with the function generator were employed for the experiment. In both the simulation and experiment, supply voltage of ±5 V was applied. The picture of the experimental setup is shown in Figure 6. The LT1228 parasitic elements were obtained from the datasheet with *I_B_* =100 μA were *R_+_* = *R*_−_ = 200 kΩ, *C_+_* = *C*_−_ = 3 pF, *R_y_* = 8 MΩ, *C_y_* = 6 pF and those obtained from the simulation were *R_T_* = 197.66 kΩ, *C_T_* = 5.95 pF, *r_x_* = 46.92 Ω, and *r_w_* = 19.80 Ω. The proposed filter was designed to obtain the *f*_0_ = 90 kHz, the pass-band gain of LP and HP was 2 (6.02 dB), and the pass-band gain of AP was unity (0 dB). Based on the ideal filtering parameter shown in Table 2, the following active and passive elements, *C* = 2.2 nF, *R*_1_ = *R_f_* = 1.2 kΩ and *I_B_* = 124.5 μA are given. Figure 7 shows the simulated and experimental results of the gain and phase responses of the HP filter by applying voltage input to node *v_in_*_1_ and connecting nodes *v_in_*_2_ and *v_in_*_3_ to ground, as indicated in Table 2. The simulated and experiment pole frequency are 87.98 kHz and 91.20 kHz, respectively. The percent errors of the simulated and experimental pole frequency were 2.24% and 1.33%, respectively. The simulated and experimental pass-band voltage gain was 1.97 (5.89 dB) and 1.99 (5.97 dB), respectively. The percent errors of the simulated and experimental pass-band gains were 1.5% and 0.5%, respectively. The simulated and experimental phase angles at the pole frequency were 44.37° and 44.59°, respectively. The percent errors of the simulated and experimental phase angles were 1.4% and 0.91%, respectively. It can be seen that the errors of the pole frequency, phase angle, and pass-band gain mostly stemmed from the parasitic elements, *C*_−_, *C_y_*, *R*_−_, and *R_y_*, as analyzed in Table 3. Moreover, the circuit accuracy at high frequency was noticeably reduced. This phenomenon is mainly caused by the LT1228 parasitic elements (especially *Z_T_*), as analyzed in Section 2.3. In the case of the experiment, this effect also stemmed from the wiring and breadboard. The simulated output impedance, *z_o_* was around 5.28 Ω.

**Figure 8:**
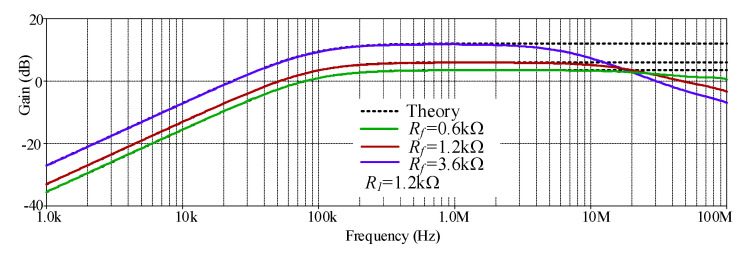
Simulated gain response of HP with different *R_f_* values.

**Figure 9:**
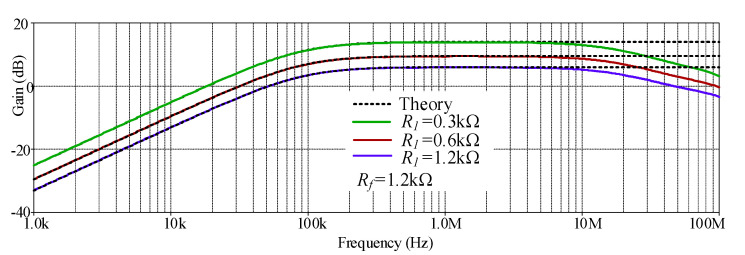
Simulated gain response of HP with different *R*_1_ values.

The simulated HP gain response with different *R_f_* values (0.6 kΩ, 1.2 kΩ, and 3.6 kΩ) is shown in Figure 8 where *R*_1_ remains at 1.2 kΩ. The simulated results revealed that the pass-band voltage gain of HP was controllable, as expected in Table 2. With these values of *R_f_*, the theoretical pass band-gains shown in Table 2 were 1.5 (3.52 dB), 2 (6.02 dB) and 4 (12.04 dB), respectively, while the simulated pass band-gains from these *R_f_* values were 1.48 (3.43 dB), 1.97 (5.91 dB), and 3.85 (11.76 dB), respectively. The percent errors of the simulated pass-band gains from these *R_f_* values were 1.33%, 1.5%, and 3.25%, respectively. The simulated result also revealed an interesting phenomenon where the HP frequency response at low value of *R_f_* gives higher bandwidth than at a high value of *R_f_*, which is consistent, as predicted in Equation (5). Thus, the value of *R_f_* should be low to obtain a higher bandwidth or operating frequency range. However, the value of *R_f_* must not be too low to cause the circuit to oscillate, as explained in [46]. The simulated HP gain response with different *R_1_* values (0.3 kΩ, 0.6 kΩ, and 1.2 kΩ) is shown in Figure 9, where *R_f_* remains at 1.2 kΩ. The simulated results revealed that the pass-band voltage gain of HP was controllable, as theoretically expected in Table 2. With these values of *R*_1_, the theoretical pass-band gains as analyzed in Table 2 were 5 (13.98 dB), 3 (9.54 dB), and 2 (6.02 dB), respectively. While the simulated pass-band gains from these *R_1_* values were 4.93 (13.86 dB), 2.96 (9.43 dB), and 1.97 (5.88 dB), respectively. The percent errors of the simulated pass-band gains from these *R*_1_ values were 1.4%, 1.33%, and 1.5%, respectively. The simulated result also revealed an interesting phenomenon where the HP bandwidths with three values of *R*_1_ were quite similar, which is consistent as predicted in Equation (5) (*R*_1_ has little impact on bandwidth). Thus, the pass-band gain should be tuned by *R*_1_ to obtain the same bandwidth.

**Figure 10:**
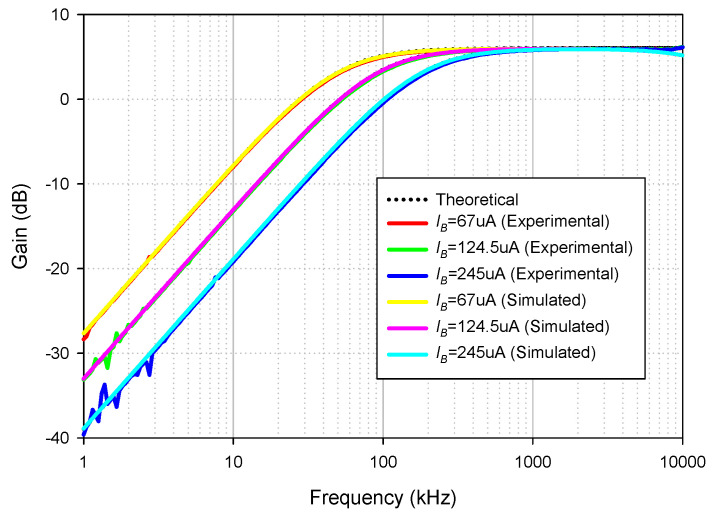
Frequency gain response of HP with different *I_B_* values.

**Figure 11:**
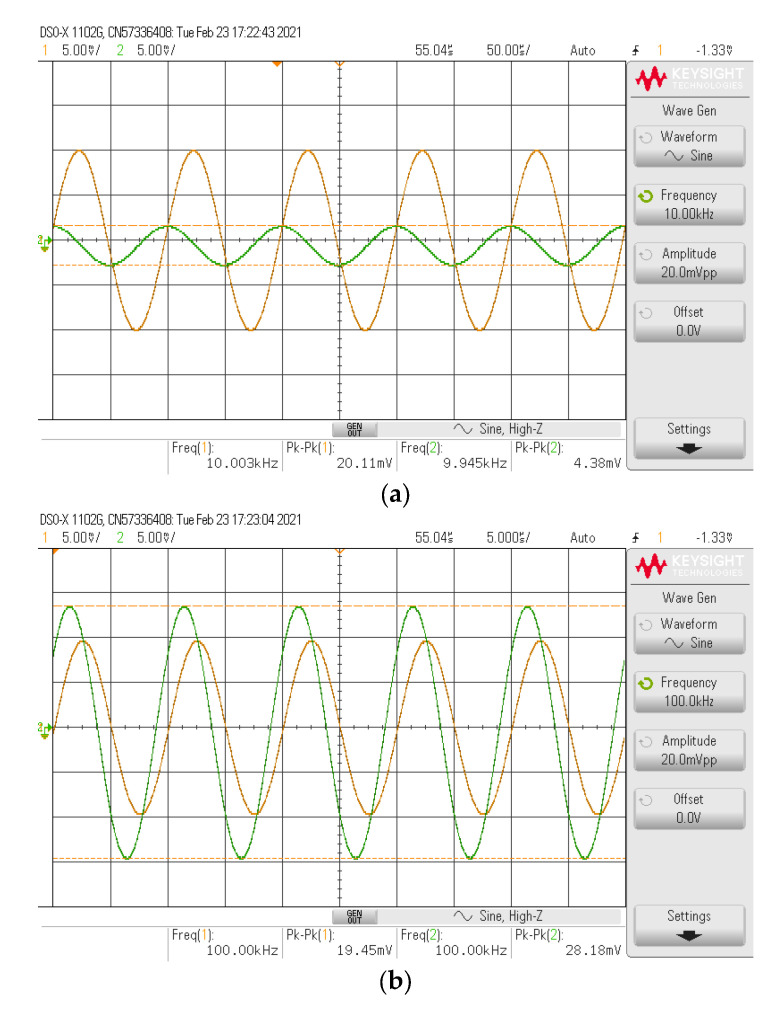

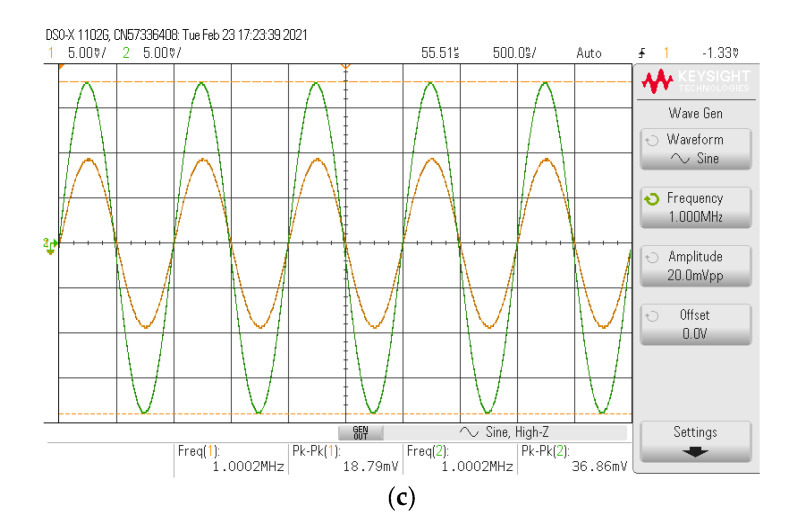
Measured input and output waveform of HP (**----***v_in_*, **-----***v_o_*) where *I_B_* = 124.5 μA. (**a**) *f* = 10 kHz. (**b**) *f* = 100 kHz. (**c**) *f* = 1 MHz.

The simulated and experimental HP gain response with different *I_B_* values (67 µA, 124.5 µA, 245 µA) are shown in Figure 10 where *R*_1_ and *R_f_* remains at 1.2 kΩ. The results revealed that the pole frequency of HP was electronically controllable, as expected in Table 2. With these values of *I_B_*, the theoretical pole frequencies calculated from *f*_0_ in Table 2 were 48.47 kHz, 90 kHz, and 177.24 kHz, respectively, while the simulated pole frequencies from these *I_B_* values were 47.7 kHz, 87.98 kHz, and 171.71 kHz, respectively. The percent errors of the simulated pole frequency from these *I_B_* values were 1.59%, 2.24%, and 3.12%, respectively. The experimental pole frequencies from these *I_B_* values were also 47.86 kHz, 91.20 kHz, and 181.97 kHz, respectively. The percent errors of the experimental pole frequency from these *I_B_* values were 1.26%, 1.33%, and 2.66%, respectively. Figure 11 shows the measured input and output waveform of HP, where the input signal amplitude was 20 mVp-p with three frequencies (10 kHz, 100 kHz, 1 MHz).

**Figure 12:**
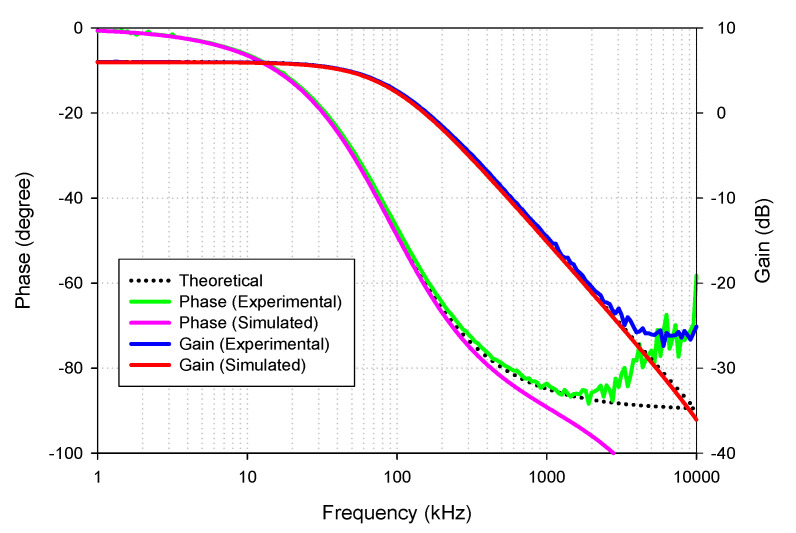
Frequency gain and phase response of LP.

Figure 12 shows the simulated and experimental results of the gain and phase responses of the LP filter by applying voltage input to node *v_in_*_2_ and connecting nodes *v_in_*_1_ and *v_in_*_3_ to ground, as indicated in Table 2. The simulated and experiment pole frequency are 87.63 kHz and 91.20 kHz, respectively. The percent errors of the simulated and experimental pole frequency were 2.63% and 1.33%, respectively. The simulated and experimental pass-band voltage gain were 1.98 (5.92 dB) and 1.99 (6.01 dB), respectively. The percent errors of the simulated and experimental pass-band gains were 1% and 0.5%, respectively. The simulated and experimental phase angles at the pole frequency were −45.76° and −44.34°, respectively. The percent errors of the simulated and experimental phase angles were 1.69% and 1.47%, respectively.

**Figure 13:**
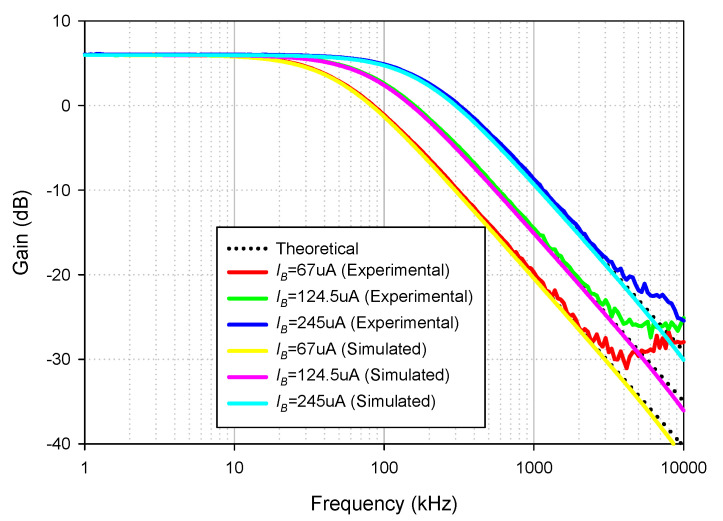
Frequency gain response of LP with different *I_B_* values.

**Figure 14:**
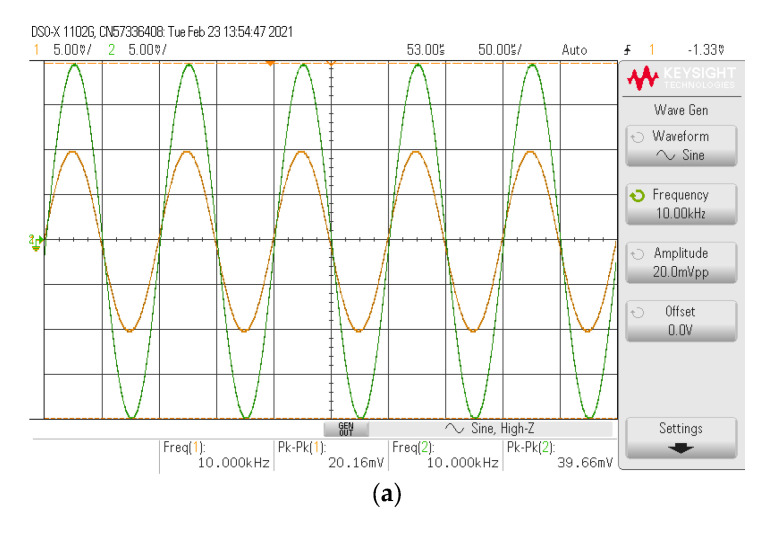

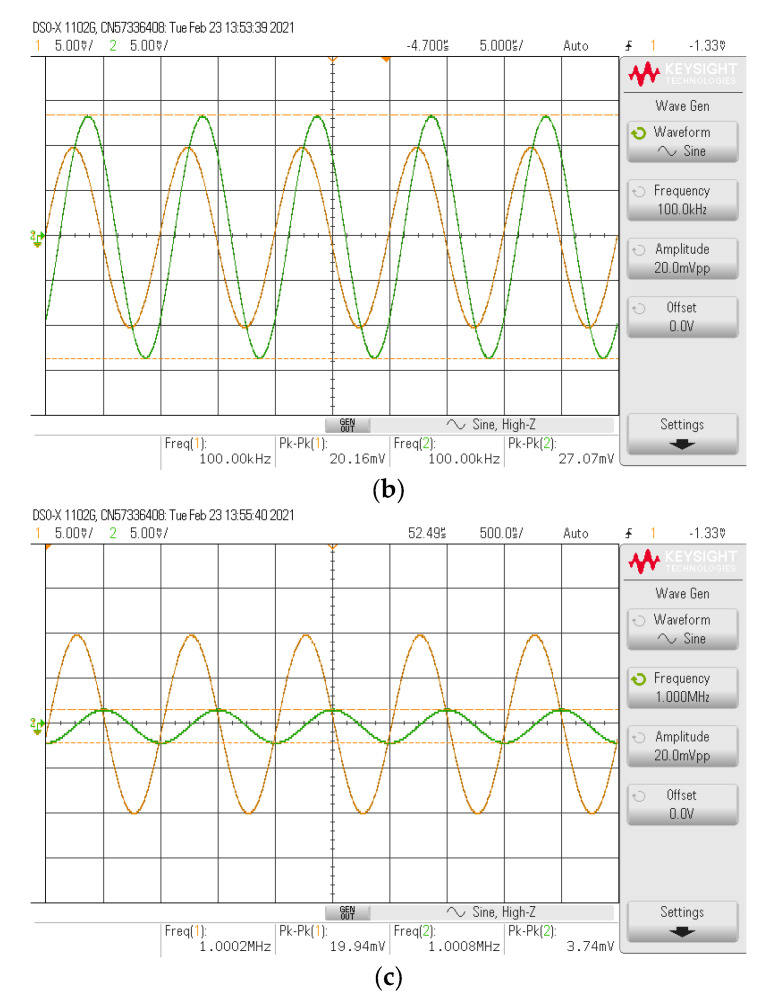
Measured input and output waveform of LP (**----***v_in_*, **-----***v_o_*) where *I_B_* = 124.5 μA. (**a**) *f* = 10 kHz. (**b**) *f* = 100 kHz. (**c**) *f* = 1 MHz.

The simulated and experimental LP gain response with different *I_B_* values (67 µA, 124.5 µA, 245 µA) are shown in Figure 13 where *R*_1_ and *R_f_* remained at 1.2 kΩ. The results revealed that the pole frequency of LP was electronically controllable, as expected in Table 2. With these values of *I_B_*, the theoretical pole frequencies were 48.47 kHz, 90 kHz, and 177.24 kHz, respectively, while the simulated pole frequencies from these *I_B_* values were 47.61 kHz, 87.63 kHz, and 170.86 kHz, respectively. The percent errors of the simulated pole frequency from these *I_B_* values were 1.77%, 2.63%, and 3.60%, respectively. The experimental pole frequencies from these *I_B_* values were also 50.11 kHz, 91.20 kHz, and 181.97 kHz, respectively. The percent errors of the experimental pole frequency from these *I_B_* values were 3.38%, 1.33%, and 2.67%, respectively. Figure 14 shows the measured input and output waveform of LP, where the input signal amplitude was 20 mVp-p with three frequencies (10 kHz, 100 kHz, 1 MHz).

**Figure 15:**
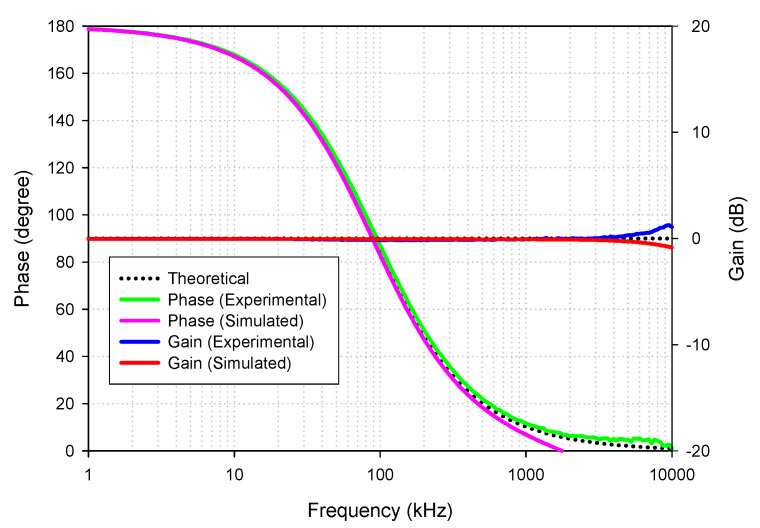
Frequency gain and phase response of AP+.

Figure 15 shows the simulated and experimental results of the gain and phase responses of the AP+ filter by applying voltage input to node *v_in_*_1_, *v_in_*_3_, and connecting nodes *v_in_*_2_ to ground, as indicated in Table 2. The result revealed that the leading phase response from 1 kHz to 10 MHz frequency changed from 180 to 0 degrees with a constant pass-band gain (0 dB), as theoretically expected in Table 2. The simulated and experimental-pass band voltage gain at the *f* = 90 kHz was 0.992 (−0.065 dB) and 0.982 (−0.15 dB), respectively. The percent errors of the simulated and experimental pass-band gains were 0.8% and 1.8%, respectively. The simulated and experimental phase angles at *f* = 90 kHz were 88.95° and 92.28°, respectively. The percent errors of the simulated and experimental phase angles were 1.17% and 2.53%, respectively.

**Figure 16:**
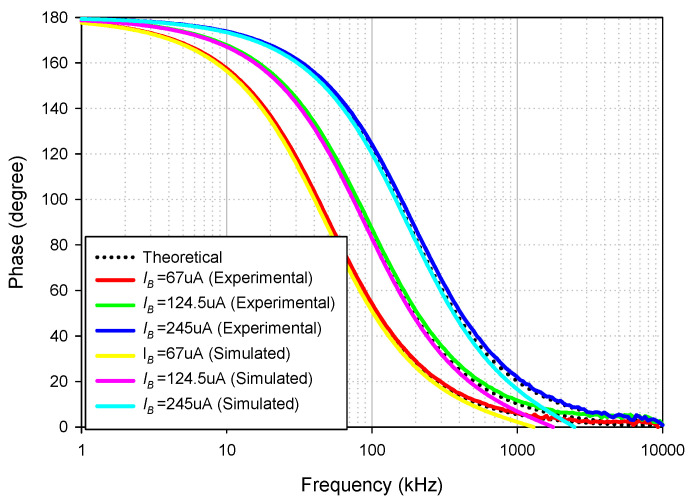
Frequency gain response of AP+ with different *I_B_* values.

**Figure 17:**
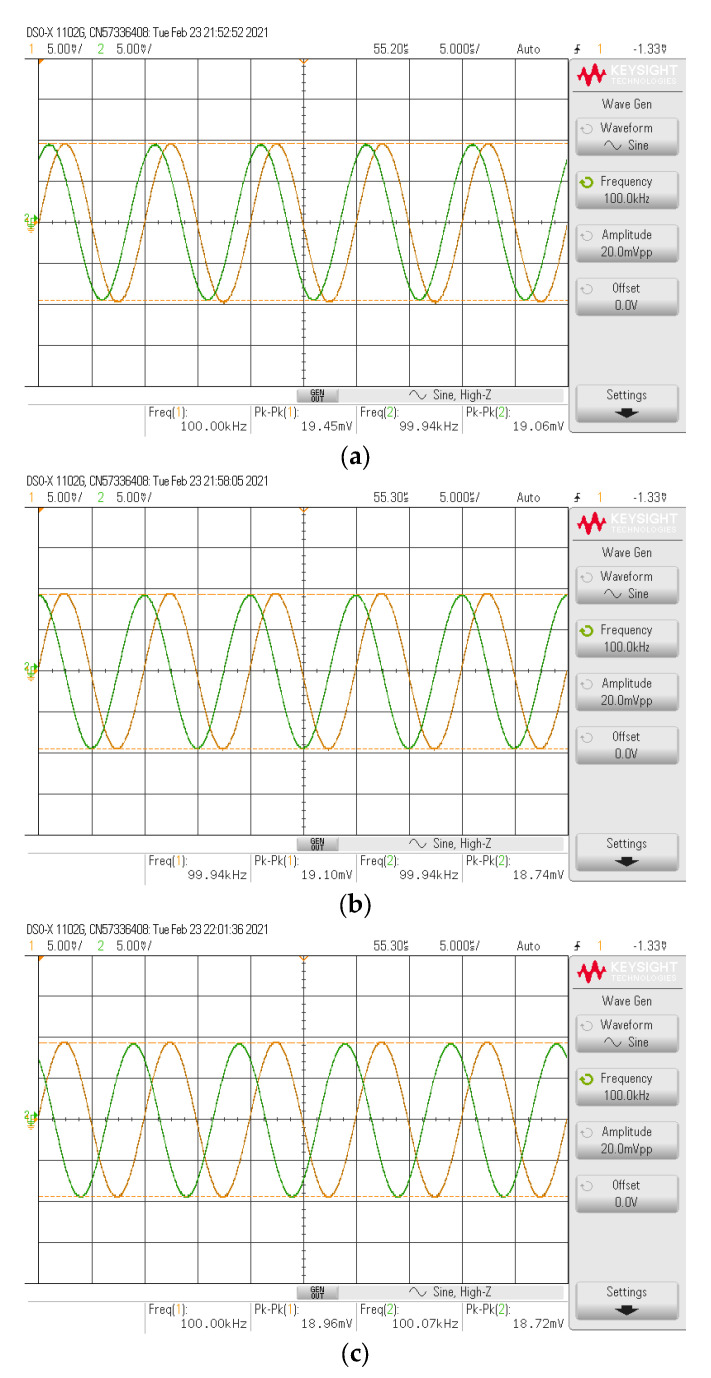
Measured input and output waveform of AP+ (**----***v_in_*, **-----***v_o_*). (**a**) *I_B_* = 67 µA. (**b**) *I_B_* = 124.5 µA. (**c**) *I_B_* = 245 µA.

The simulated and experimental AP+ phase response with different *I_B_* values (67 µA, 124.5 µA, 245 µA) is shown in Figure 16 where *R*_1_ and *R_f_* remained at 1.2 kΩ. The results revealed that the phase of AP+ was electronically controllable, as expected in Table 2. With these values of *I_B_*, the theoretical phase angles at *f* = 90 kHz were 56.62°, 90°, and 126.15°, respectively, while the simulated phase angles from these *I_B_* values were 55.86°, 88.95°, and 125.26°, respectively. The percent errors of the simulated phase angles from these *I_B_* values were 1.34%, 1.17%, and 0.71%, respectively. The experimental phase angles from these *I_B_* values were also 58.54°, 92.28°, and 128.49°, respectively. The percent errors of the experimental phase angles from these *I_B_* values were 3.39%, 2.53%, and 1.85%, respectively. Figure 17 shows the measured input and output waveform of AP+ with different *I_B_* values (67 µA, 124.5 µA, 245 µA) where the input signal amplitude was 20 mVp-p.

**Figure 18:**
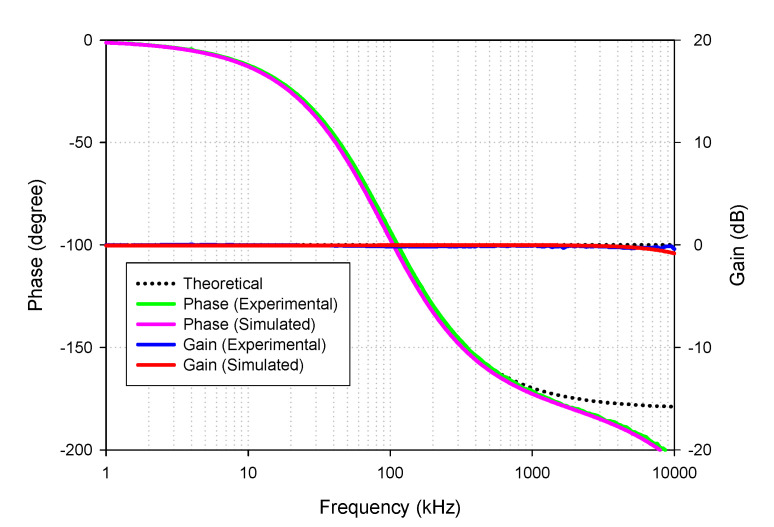
Frequency gain and phase response of AP−.

Figure 18 shows the simulated and experimental results of the gain and phase responses of the AP− filter by applying the voltage input to node *v_in_*_2_, *v_in_*_3_, and connecting nodes *v_in_*_1_ to ground, as indicated in Table 2. The result revealed that the lagging phase response from 1 kHz to 10 MHz frequency changed from 0 to −180 degrees with a constant pass-band gain (0 dB) as theoretically expected in Table 2. The simulated and experimental pass-band voltage gain at *f* = 90 kHz was 0.995 (−0.037 dB) and 0.982 (−0.15 dB), respectively. The percent errors of the simulated and experimental pass-band gains were 0.5% and 1.8%, respectively. The simulated and experimental phase angles at *f* = 90 kHz were −91.18° and −87.97°, respectively. The percent errors of the simulated and experimental phase angles were 1.31% and 2.25%, respectively.

**Figure 19:**
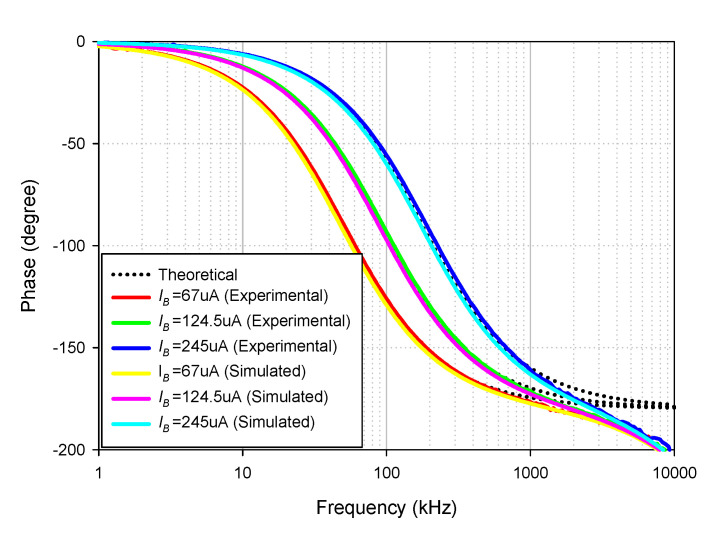
Frequency gain response of AP− with different *I_B_* values.

The simulated and experimental AP− phase response with different *I_B_* values (67 µA, 124.5 µA, 245 µA) is shown in Figure 19 where *R*_1_ and *R_f_* remained at 1.2 kΩ. The results revealed that the phase of AP− was electronically controllable, as expected in Table 2. With these values of *I_B_*, the theoretical phase angles at *f* = 90 kHz were −126.15°, −90°, and −56.62°, respectively. At the same time, the simulated phase angles from these *I_B_* values were −124.22°, −91.18°, and −54.6°, respectively. The percent errors of the simulated phase angles from these *I_B_* values were 1.53%, 1.31%, and 3.56%, respectively. The experimental phase angles from these *I_B_* values were also −121.63°, −87.97°, and −51.51°, respectively. The percent errors of the experimental phase angles from these *I_B_* values were 3.58%, 2.25%, and 9.02%, respectively.

**Figure 20:**
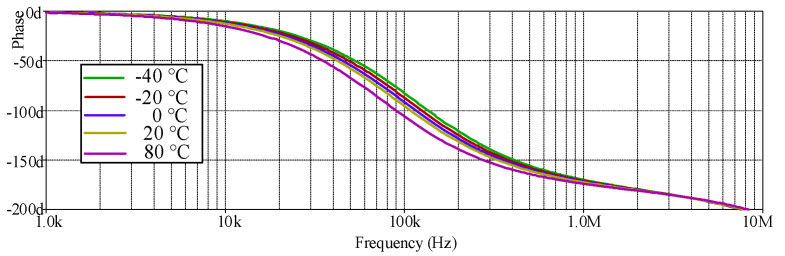
Frequency gain response of AP− with different temperature values.

**Figure 21:**
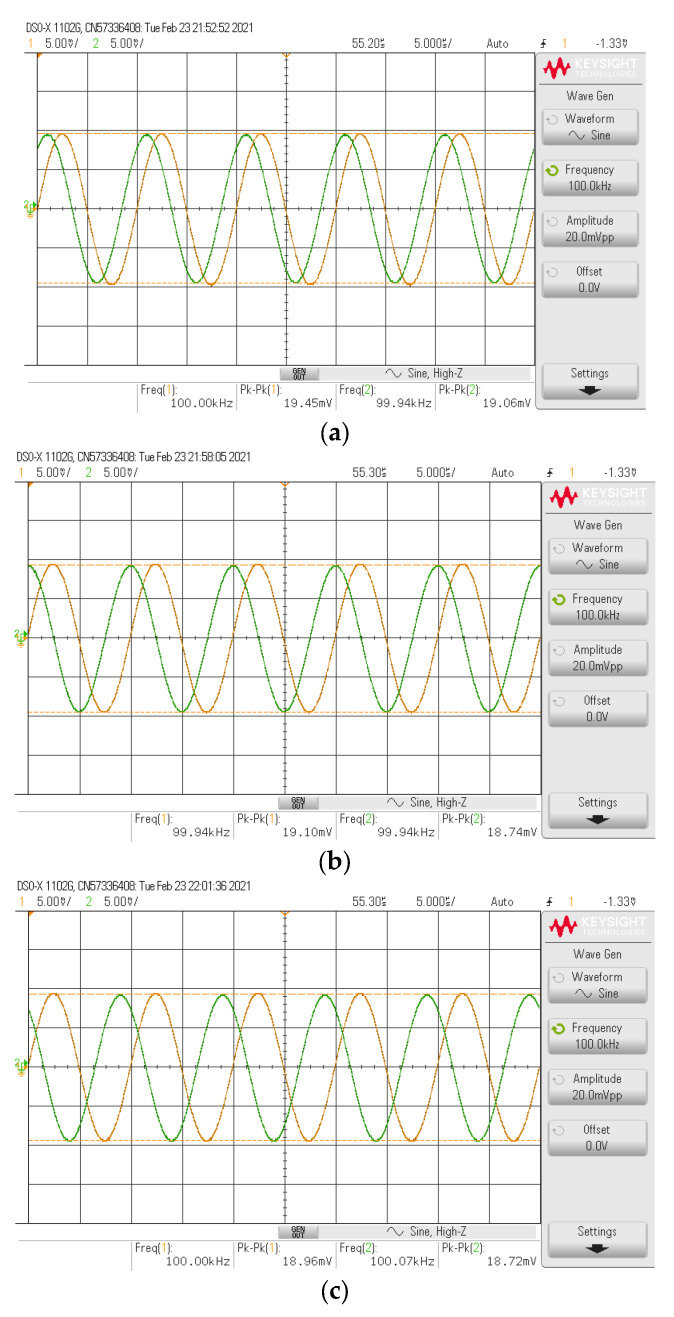
Measured input and output waveform of AP− (**----***v_in_*, **-----***v_o_*). (**a**) *I_B_* = 67 µA. (**b**) *I_B_* = 124.5 µA. (**c**) *I_B_* = 245 µA.

The simulated AP− phase response with different temperature values (−40 °C, −20 °C, 0 °C, 20 °C, and 80 °C) is shown in Figure 20 where *R_1_* and *R_f_* remained at 1.2 kΩ, *I_B_* = 124.5 µA. The simulated results revealed that the temperature had little impact on the phase response of the proposed circuit, as expected in Table 2. Figure 21 shows the measured input and output waveform of AP− with different *I_B_* values (67 µA, 124.5 µA, 245 µA) where the input signal amplitude was 20 mV_p-p_.

**Figure 22:**
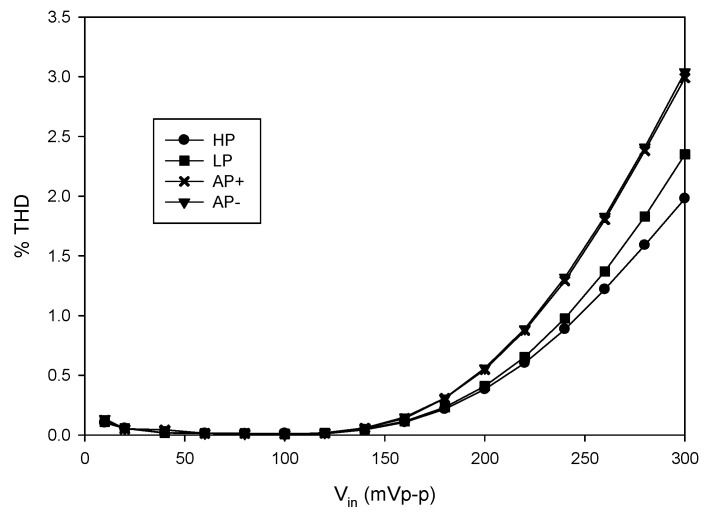
THD variations with input voltage signal at 90 kHz.

**Figure 23:**
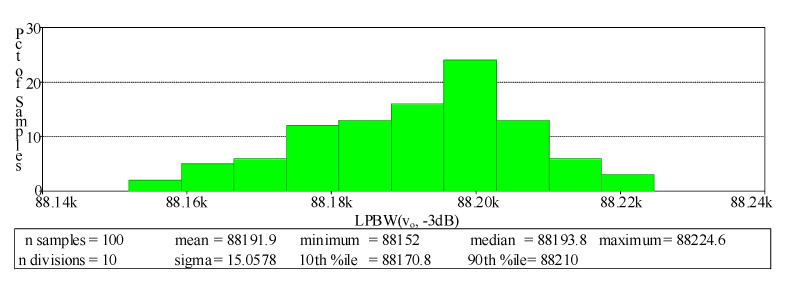
Histogram of the Monte Carlo analysis with a 10% deviation in *β_F_* for LP filtering function.

Table 4 summarizes the filtering verification where *C* = 2.2 nF, *R*_1_ = *R_f_* = 1.2 kΩ and *I_B_* = 124.5 μA. Since the simplified structure of the commercially available IC, LT1228, shows that the *g_m_* stage is based on bipolar folded cascode OTA, then the third harmonic distortion can be calculated as follows [47]:

(7) $$HD_{3}=\frac{1}{48}{\left(\frac{v_{id}}{V_{T}}\right)}^{2}$$

where *v_id_* is the differential input voltage (*v_id_* = *v_+_*-*v*_−_). Therefore, the total harmonic distortion (THD) of the output voltage against the amplitude of input voltage for each filtering function was tested to evaluate the input dynamic range of the proposed filter. The THD dependence on the amplitude of the input voltage signal obtained from the simulation is shown in Figure 22 where the frequency of the sinusoidal input signal was 90 kHz. The THD of all filtering functions was below 1%, where the input volage was lower than 200 mVp-p. In order to examine the changes of the forward beta (*β_F_*) for all the NPN and PNP transistors in LT1228 affecting the circuit performances, the Monte Carlo analysis of the proposed LP filter was carried out. In this simulation, the value of *β_F_* varied by 20% Gaussian deviation, while other active and passive elements were the same as stated above. After 100 runs, the magnitude and phase responses of the input impedance of the proposed capacitance multiplier are shown in Figure 23.

## 4. Quadrature Sinusoidal Oscillator Based on the Proposed AP− Filter

**Figure 24:**
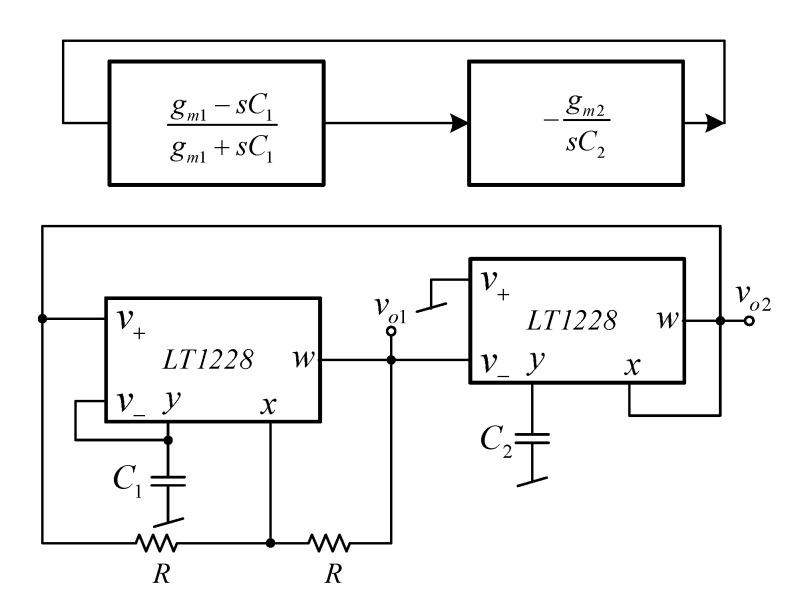
Proposed quadrature sinusoidal oscillator.

The proposed inverting first-order all-pass (AP−) filter was selected as the sub-circuit to design the quadrature oscillator. By cascading the AP−filter with the inverting lossless integrator, as shown in Figure 24, the voltage-mode quadrature sinusoidal oscillator is obtained. A straightforward circuit analysis yielded the following characteristic equation

(8) $$s^{2}C_{1}C_{2}+sC_{1}\left(g_{m2}-g_{m1}\right)+g_{m1}g_{m2}=0.$$

From Equation (8), the expression of the frequency of oscillation (FO) and the condition of oscillator (CO) are given by

(9) $$\omega_{0}=\sqrt{\frac{g_{m1}g_{m2}}{C_{1}C_{2}}}=10\sqrt{\frac{I_{B1}I_{B2}}{C_{1}C_{2}}}.$$

and

(10) $$g_{m1}\leq g_{m2};\;I_{B1}\leq I_{B2}.$$

From Equations (9) and (10), the FO and CO can be electronically tuned by *I_B_*_1_ and *I_B_*_2_. The 90° phase difference will occur between two output voltages, *v_o_*_1_ and *v_o_*_2_, where phase of *v_o_*_2_ leads to phase *v_o_*_1_. Note that the output voltage nodes are low impedance, so extra voltage buffers are not required.

**Figure 25:**
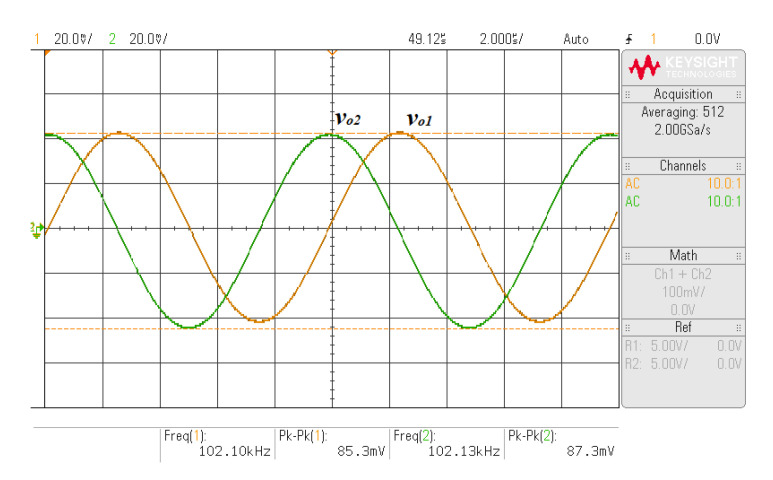
Measured quadrature waveform.

**Figure 26:**
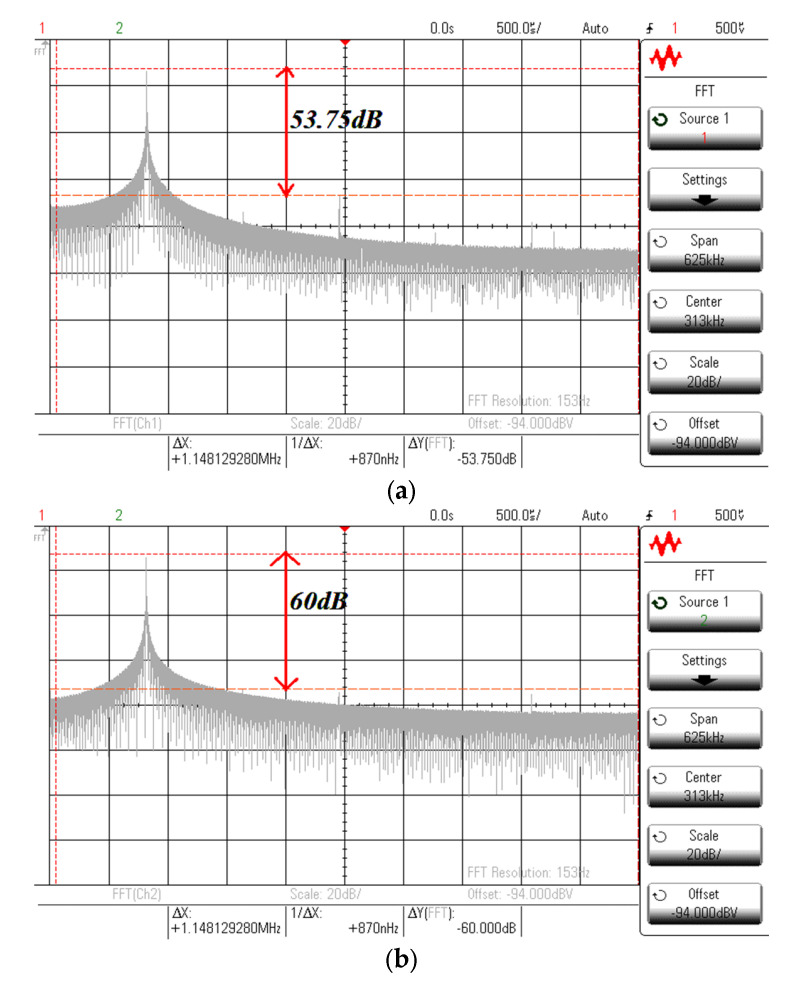
Measured output spectrum of *v_o_*_1_ and *v_o_*_2_. (**a**) *v_o_*_1_. (**b**) *v_o_*_2_.

To verify the functionality of the proposed quadrature oscillator in Figure 24, the experiment using the commercially available LT1228 IC was carried out. The supply voltage of ±5 V was applied with *C*_1_ = *C*_2_ = 2.2 nF, *R* = 1 kΩ, *I_B_*_1_ = 132.58 μA, and *I_B_*_2_ = 137.41 μA. The 470 Ω resistor was connected between the *x* and *w* terminal of second LT1228 to avoid the oscillation at other frequencies. Figure 25 shows the measured quadrature waveform. Figure 26a,b shows the output spectrum of *v_o_*_1_ and *v_o_*_2_, respectively.

## 5. Conclusions

In this paper, the voltage-mode multifunction first-order filter built with single commercially available LT1228 IC was proposed. It can provide four first-order filtering responses. For the all-pass functions employed as the phase shifter, both lagging and leading phase responses were obtained with the same configuration. The pole frequency and phase shift angle were electronically controlled via bias current *I_B_*. Although the proposed all-pass functions require the matching conditions of *R*_1_ and *R_f_*, tuning of the lagging and leading phase responses can be adjusted from the single *I_B_* without matching conditions from two or three parameters during the tuning process. The pass-band gain of HP and LP filtering function was controllable via *R*_1_ and *R_f_*. The analysis of LT1228 parasitic effects revealed that the *Z_T_* was the most effective in filtering performances. To reduce the effect of *Z_T_*, the value of *R_f_* should be low. At low value of *R_f_*, the bandwidth of the proposed multifunction filter could also be enhanced. Therefore, the pass-band gain of the LP and HP filter should be tuned via *R*_1_ without affecting the bandwidth. The performances of the proposed filter were verified through the simulation and experiment. The simulation and experimental results confirm the theoretical propositions. Moreover, the quadrature sinusoidal oscillator realized from the proposed first-order all-pass filter is shown as the application example.

## Figures and Tables

**Table 1:** Comparison of the proposed design with previous first-order multifunction filters.

Ref.	Mode	Number of ABB	Commercially Available IC	R + C	No use of Multiple Output ABB	Functions	Gain Controllability	Electronic Controllable	Voltage Supplies & Power Dissipation	Zero/Pole Frequency Adjustable Simultaneously by Single Parameter	Pole Frequency (Hz)	Cascade-Able at Output Node
[21]	CM	2 ICCII & 1 MOS	Yes (7 AD844)	0 + 1	No	LP, HP, AP−	No	Yes *	±0.75 V & 3.29 mW	Yes	7.96 M	Yes
[22] Figure 2	RM	2 CVCII	No	2 + 1	Yes	LP, AP−	LP, AP	Yes	±0.9 V & 0.385 mW to 1.057 mW	Yes	89 k–1 M	Yes
[22] Figure 3	RM	2 CVCII	No	2 + 1	Yes	LP, HP	LP, HP	Yes		Yes	89 k–1 M	Yes
[23] Figure 2	CM	2 CCII	No	1 + 1	No	LP, HP, AP+	No	No	±1.25 V & 3.71 mW	Yes	15.9 M	Yes
[23]	VM	2 DDCC	No	1 + 1	Yes	LP, HP, AP+	No	No	±1.25 V & NA	Yes	15.9 M	Yes
[24]	CM	1 DDDXCCII & 4 MOS	No	0 + 1	No	LP, HP, AP−	No	Yes *	±1.25 V & 2 mW	No	3 M	Yes
[25]	VM	1 OTRA	No	2 + 2	Yes	LP, HP, AP−	LP, HP	No	±1.5 V & NA	No	100 k	Yes
[26]	CM	1 EX-CCCII	No	0 + 1	No	LP, HP, AP+	No	Yes	±1.25 V & 0.44 mW to 4.4 mW	Yes	3.93 M	Yes
[27]	VM	2 OTA	Yes	1 + 1	Yes	LP, HP, AP−	HP	Yes	±0.4 V & 47.2 μW	Yes	8.05 k	No
[28]	TM	1 CCDDCCTA	No	0 + 1	Yes	LP, HP, AP+	LP, HP, AP+	Yes	±0.9 V & NA	Yes	1.24 M	Yes
[29]	CM	1 DDDXCCII	No	3 + 1	No	LP, HP, AP−	No	No	±1.2 V & NA	No	6.43 M	Yes
[30]	CM	1 DXCCTA	Yes (4 AD844, 1 LM13700)	0 + 2	No	LP, HP, AP−	No	Yes	±1.25 V & 1.75 mW	No	10 M	Yes
[31] Figure 1	CM	1 MO-DXCCTA	Yes (4 AD844, 2 LM13700)	0 + 2	No	LP, HP, AP−	No	Yes	±1.25 V & 1.38 mW	Yes	11.7 M	Yes
[31] Figure 2	TM	1 MO-DXCCTA	Yes (4 AD844, 2 LM13700)	0 + 2	No	LP, HP, AP−	LP, HP, AP	Yes	±1.25 V & 1.4 mW	Yes	11.7 M	Yes
[32]	VM	2 subtractor	Yes (4 AD844)	1 + 1	Yes	LP, HP, AP+, AP−	No	No	±0.75 V & 1.77 mW	Yes	6.37 M	Yes (HP & AP)
[33]	VM	1 M-CCCCTA	No	1 + 1	Yes	LP, HP, AP−	HP	Yes	±2.5 V & NA	Yes	286.21 k	No
[34]	CM	1 DX-MOCCII	No	1 + 1	No	LP, HP, AP+, AP−	No	No	±0.75 V & 2.75 mW	Yes	7.96 M	Yes
[35]	CM	2 ICCII & 1 MOS	No	0 + 1	No	LP, HP, AP−, AP+	No	Yes *	±0.75 V & 4.08 mW	Yes	2.6 M	Yes
[36]	CM	2 DO-CCII	No	1 + 1	No	LP, HP, AP+	No	Yes	±5 V & 25.7 mW	Yes	6.36 M	Yes
[37]	CM	3 PCA	No	1 + 1	No	LP, HP, AP+, AP−	LP, HP, AP+, AP−	Yes	±5 V & NA	Yes	100 k	Yes
[38]	VM	1 ZC-CCCFDTA & 1 CA	Yes (1 AD830, 1 VCA610, 1 EL4083, 2 OPA660)	1 + 1	No	HP, AP+, AP−	No	Yes	±1.5 V & NA	Yes	339 k	No
[39]	CM	1 CFTA	No	0 + 1	No	LP, HP, AP+, AP−	No	Yes	±1.5 V & NA	Yes	NA	Yes
[40]	VM	3 OTRA	No	6 + 3	Yes	LP, HP, AP+	No	No	±1.25 V & NA	No	100 k	Yes
[41]	CM	2 CCII	No	2 + 1	No	LP, HP, AP+	No	No	NA	No	1.326 M	Yes
[42]	VM	2 CCII	Yes (3 AD844)	4 + 1	Yes	LP, HP, AP+	LP	No	NA	Yes	200 k	No
[43]	VM	1 LT1228	Yes	6 + 1	Yes	LP, HP, AP−	LP, HP	Yes	NA	Yes	100 k	Yes
[44]	VM	1 VD-DIBA	Yes (1 LT1228, 1 AD830	2 + 1	Yes	LP, HP, AP−, AP+	LP, HP	Yes	±5 V & NA	Yes	159.15 k	Yes
[45]	CM	2 DVCC	Yes (10 AD844)	1 + 1		LP, HP, AP−, AP+	No	No	±1.25 V & 3.65 mW	Yes	1.99 M	Yes
This work	VM	1 LT1228	Yes	2 + 1	Yes	LP, HP, AP−, AP+	LP, HP	Yes	±5 V & 57.6 mW	Yes	90 k	Yes

* [21,24,35] The passive resistor is replaced by the MOS transistor to be achieved the electronic controllability. * RM is resistance mode (current is input and voltage is output); TM is transconductance mode (voltage is input and current is output), NA is the information not available. * [28] needs additional inverting unity gain amplifier. The voltage supplies, power consumption and frequency range are obtained from the simulation.

**Table 2:** The selection of each filtering response and filtering parameters.

Input			Transfer Function	Filtering Function	Pass-Band Gain	Phase Response	Pole Frequency
*v_in_*_1_	*v_in_*_2_	*v_in_*_3_					
1	0	0	$\frac{\left(1+\frac{R_{f}}{R_{1}}\right)s}{s+\frac{g_{m}}{C}}$	High-pass (HP)	$\frac{R_{f}}{R_{1}}+1$	$90-\tan^{-1}\frac{\omega C}{g_{m}}$ $\mathrm{or}\;90-\tan^{-1}\frac{3.87V_{T}\omega C}{I_{B}}$	$\omega_{0}=\frac{g_{m}}{C}$ $\mathrm{or}\;\omega_{0}=\frac{I_{B}}{3.87V_{T}C}$
0	1	0	$\frac{\left(1+\frac{R_{f}}{R_{1}}\right)\frac{g_{m}}{C}}{s+\frac{g_{m}}{C}}$	Low-pass (LP)	$\frac{R_{f}}{R_{1}}+1$	$-\tan^{-1}\frac{\omega C}{g_{m}}$ $\mathrm{or}-\tan^{-1}\frac{3.87V_{T}\omega C}{I_{B}}$	
1	0	1	$\frac{s-\frac{g_{m}}{C}}{s+\frac{g_{m}}{C}}$ $\mathrm{where}\;\frac{R_{f}}{R_{1}}=1$	Non-inverting all-pass (AP+)	1	$180-2\tan^{-1}\frac{\omega C}{g_{m}}$ $\mathrm{or}\;180-2\tan^{-1}\frac{3.87V_{T}\omega C}{I_{B}}$	
0	1	1	$\frac{-\left(s-\frac{g_{m}}{C}\right)}{s+\frac{g_{m}}{C}}$ $\mathrm{where}\;\frac{R_{f}}{R_{1}}=1$	Inverting all-pass (AP−)	1	$-2\tan^{-1}\frac{\omega C}{g_{m}}$ $\mathrm{or}-2\tan^{-1}\frac{3.87V_{T}\omega C}{I_{B}}$	

**Table 3:** The filtering parameters with parasitic effects.

Filtering Function	Transfer Function	Pass-Band Gain	Phase Response	Pole Frequency
High-pass	$\frac{s\frac{C}{g_{m}}\left(\frac{R_{f}}{R_{1}}+1\right)}{s\frac{C^{*}}{g_{m}}+\frac{1}{g_{m}R^{*}}+1}$	$\frac{C}{C^{*}}\left(1+\frac{R_{f}}{R_{1}}\right)$	$90-\tan^{-1}\frac{\omega C^{*}}{g_{m}\left(\frac{1}{g_{m}R^{*}}+1\right)}$	$\omega_{0}^{*}=\frac{1}{C^{*}R^{*}}+\frac{g_{m}}{C^{*}}$
Low-pass	$\frac{\left(\frac{R_{f}}{R_{1}}+1\right)}{s\frac{C^{*}}{g_{m}}+\frac{1}{g_{m}R^{*}}+1}$	$\left(1+\frac{R_{f}}{R_{1}}\right)\left(\frac{g_{m}}{\frac{1}{R^{*}}+g_{m}}\right)$	$-\tan^{-1}\frac{\omega C^{*}}{g_{m}\left(\frac{1}{g_{m}R^{*}}+1\right)}$	
Non-inverting all-pass	$\frac{s\frac{C^{**}}{g_{m}}-\frac{1}{g_{m}R^{*}}-1}{s\frac{C^{*}}{g_{m}}+\frac{1}{g_{m}R^{*}}+1}$ where $\frac{R_{f}}{R_{1}}=1$	$\sqrt{\frac{{\left(\omega \frac{C^{**}}{g_{m}}\right)}^{2}+{\left(\frac{1}{g_{m}R^{*}}+1\right)}^{2}}{{\left(\omega \frac{C^{*}}{g_{m}}\right)}^{2}+{\left(\frac{1}{g_{m}R^{*}}+1\right)}^{2}}}$	$180-\left\{\begin{array}{l} \tan^{-1}\left[\omega \left(\frac{C^{**}}{g_{m}+\frac{1}{R^{*}}}\right)\right]-\\ \tan^{-1}\left[\omega \left(\frac{C^{*}}{g_{m}+\frac{1}{R^{*}}}\right)\right] \end{array}\right\}$	
Inverting all-pass	$\frac{-s\frac{C^{*}}{g_{m}}-\frac{1}{g_{m}R^{*}}+1}{s\frac{C^{*}}{g_{m}}+\frac{1}{g_{m}R^{*}}+1}$ where $\frac{R_{f}}{R_{1}}=1$	$\sqrt{\frac{{\left(\omega \frac{C^{*}}{g_{m}}\right)}^{2}+{\left(1-\frac{1}{g_{m}R^{*}}\right)}^{2}}{{\left(\omega \frac{C^{*}}{g_{m}}\right)}^{2}+{\left(1+\frac{1}{g_{m}R^{*}}\right)}^{2}}}$	$\left\{\begin{array}{l} -\tan^{-1}\left[\omega \left(\frac{C^{*}}{g_{m}-\frac{1}{R^{*}}}\right)\right]-\\ \tan^{-1}\left[\omega \left(\frac{C^{*}}{g_{m}+\frac{1}{R^{*}}}\right)\right] \end{array}\right\}$	

where $C^{*}=C+C_{-}+C_{y}$; $C^{**}=C-C_{-}-C_{y}$; $R^{*}=R_{-}∥R_{y}$.

**Table 4:** The characteristics of the proposed multifunction filter.

Filtering Function	Pole Frequency (kHz)			Pass-Band Gain (dB)			Phase Response (Degree)		
	Expect	Simulation	Experiment	Expect	Simulation	Experiment	Expect	Simulation	Experiment
High-pass	90	87.98	91.20	6.02	5.89	5.97	45	44.37°	44.59°
Low-pass	90	87.63	91.20	6.02	5.92	6.01	−45	−45.76°	−44.34°
Non-Inverting all-pass	90	88.73	95.45	0	−0.065	−0.15	90	88.95°	92.28°
Inverting all-pass	90	88.57	95.45	0	−0.037	−0.15	−90	−91.18°	−87.97°
